# Different Capacity of Monocyte Subsets to Phagocytose Iron-Oxide Nanoparticles

**DOI:** 10.1371/journal.pone.0025197

**Published:** 2011-10-03

**Authors:** Marcus Settles, Martin Etzrodt, Katja Kosanke, Matthias Schiemann, Alexander Zimmermann, Reinhard Meier, Rickmer Braren, Armin Huber, Ernst J. Rummeny, Ralph Weissleder, Filip K. Swirski, Moritz Wildgruber

**Affiliations:** 1 Institut für Radiologie, Klinikum Rechts der Isar, Technische Universität München, Munich, Germany; 2 Center for Systems Biology, Massachusetts General Hospital and Harvard Medical School, Boston, Massachusetts, United States of America; 3 Institut für medizinische Mikrobiologie, Immunologie und Hygiene, Klinikum Rechts der Isar, Technische Universität München and Clinical Cooperation Group “Antigen-Specific Immunotherapy”, Helmholtzzentrum München, Munich, Germany; 4 Klinik und Poliklinik für Gefäßchirurgie, Klinikum Rechts der Isar, Technische Universität München, Munich, Germany; University of Lyon, France

## Abstract

**Objective:**

To explore the capacity of human CD14^+^CD16^++^ and CD14^++^CD16^-^ monocytes to phagocyte iron-oxide nanoparticles in vitro.

**Methods:**

Human monocytes were labeled with four different magnetic nanoparticle preparations (Ferumoxides, SHU 555C, CLIO-680, MION-48) exhibiting distinct properties and cellular uptake was quantitatively assessed by flow cytometry, fluorescence microscopy, atomic absorption spectrometry and Magnetic Resonance Imaging (MRI). Additionally we determined whether cellular uptake of the nanoparticles resulted in phenotypic changes of cell surface markers.

**Results:**

Cellular uptake differed between the four nanoparticle preparations. However for each nanoparticle tested, CD14^++^CD16^-^ monocytes displayed a significantly higher uptake compared to CD14^+^CD16^++^ monocytes, this resulted in significantly lower T1 and T2 relaxation times of these cells. The uptake of iron-oxide nanoparticles further resulted in a remarkable shift of expression of cell surface proteins indicating that the labeling procedure affects the phenotype of CD14^+^CD16^++^ and CD14^++^CD16^-^ monocytes differently.

**Conclusion:**

Human monocyte subsets internalize different magnetic nanoparticle preparations differently, resulting in variable loading capacities, imaging phenotypes and likely biological properties.

## Introduction

Monocytes originate from the bone marrow, and are released into the circulation where they can circulate several days until they extravasate and populate healthy and diseased tissues [1]. Upon extravasation monocytes can differentiate and serve as a source to replenish tissue resident macrophages and dendritic cells [2]. As mediators both of innate and adaptive immunity they are involved in tissue homeostasis and various diseases such as bacterial and viral infections, cancer and atherosclerosis [2]. In order to study various disease mechanisms by means of biomedical imaging, ex vivo labeling of monocytes with various agents and subsequent tracking of these cells has been performed [3]. Additionally, in vivo phagocytosis of various nanoparticle imaging agents has been utilized to characterize disease stages, e.g. to discriminate between benign or malignant neoplastic lesions [4], [5] or to track macrophage infiltration in autoimmune encephalitis and multiple sclerosis [6], [7]. Superparamagnetic iron-oxide based nanoparticles are the most extensively studied materials used for either non-specific labeling or specific targeting of cell surface receptors via high-affinity ligands on functionalized nanoparticles [8]. Biocompatible magnetic nanoparticles are often classified by composition, size, coating, crystallinity and manufacturing process. Preparations <100nm are often lumped as ultrasmall SPIO (USIPO) and single crystal nanoparticles as monocrystalline iron-oxide nanoparticles (MION). Iron-only nanoparticles contain both Fe^2+^ and Fe^3+^ ions in variable stochiometric ratios, e.g. Fe_2_^3+^O_3_Fe^2+^O [9]. To keep the particles suspended, the core is surrounded by various coating materials such as dextrans, modified dextrans or other polymers.

Magnetic nanoparticles shorten both the T1 and T2/T2* relaxation with subsequent signal decrease in Magnetic Resonance Imaging (MRI). The effects on signal intensity depend on particle composition, particle size, concentration of particles within a given imaging voxel and signal acquisition parameters. The magnetic field strength has a non-linear influence on the obtained signal, however with minor importance in clinical practice. Metz et al. have analyzed the phagocytosis of various approved iron-oxide contrast agents by human monocytes and optimized labeling protocols [10]. Similarly Oude Engeberink et al. performed labeling of freshly isolated human monocytes with SPIOs and USPIOs, but also investigated further distinct activation patterns as well as functional parameters after the labeling procedure [11]. However in both studies, labeling of monocytes has been conducted in an unselective manner with no regard to different monocyte subpopulations [10], [11].

Monocyte heterogeneity is conserved in humans and mice [1], [12], [13]. In the mouse, at least two monocyte subpopulations have been identified with divergent functional properties [14], [15]. While Ly6C^++^CD43^+^ monocytes have been found to propagate disease and promote inflammation, Ly6C^+^CD43^++^ monocytes attenuated inflammation and are involved in tissue repair. Similarly in humans, at least two principal monocyte subsets have been identified that can be distinguished by the expression of CD 14 and CD16: CD14^++^CD16^-^ monocytes and CD14^+^CD16^++^ monocytes. Although some similarities have been identified between mice and humans [12], a possible homology of the described subsets between the different species is not clear yet. In humans, elevated serum levels of CD14^+^CD16^++^ have been observed in coronary artery disease, sepsis, HIV infection, kidney failure and cancer [2], [16], [17], [18], [19], [20], [21]. The increasing evidence that monocyte subsets play divergent roles in various human diseases has not been taken into account in studies involving cell labeling and tracking with MRI. We have previously shown that monocyte subsets have different phagocytosis capacity for macromolecules and magnetic nanoparticles [8]. In this study we investigate magnitude and effects of cellular iron-oxide nanoparticle uptake by flow cytometry, fluorescence microscopy, atomic absorption spectrometry and MR imaging. Using the most salient nanomaterial with the highest native cellular uptake, we perform selective phenotyping experiments to assess possible alterations of cell surface proteins.

## Materials and Methods

### Isolation of human monocytes

The study protocol was approved by the institutional review board at Harvard Medical School, Boston, MA. The institutional review board specifically approved the use of human blood specimens for the performed ex-vivo experiments. Whole blood was obtained from healthy volunteers (8 male, 7 female, age 37±4 years). All donors gave written and informed consent. Fresh whole blood was drawn into heparinized collection tubes. To obtain leukocyte suspensions, whole blood was diluted 1∶1 with Dulbecoo's Phosphate Buffered Saline (DPBS) and 20 ml diluted blood was overlaid on a 15 ml density gradient (Ficoll-Paque Plus, density 1.077 g/ml, GE Healthcare, NJ) and centrifuged (20 min, 1600 rpm, 18°C). The mononuclear cell interphase was carefully isolated and washed 3 times with DPBS. Resuspended cell suspensions were counted using Trypan blue (Cellgro, Mediatech Inc., Manassas, VA).

### Phagocytosis assay

For the determination of differences in phagocytosis between both monocyte subsets, yellow-green labeled latex beads were used (Bead size 2.0 µm, Sigma, Saint Louis, MO). FACS-sorted monocyte subsets where incubated at a cell/bead ratio of 1/25 (10^6^ cells in 1ml final volume) for 4h at 37°C in RPMI 1640, supplemented with 1% Penicillin/Streptomycin and 10% heat-inactivated fetal calf serum (FCS). After incubation, free beads were washed from the cell suspension 3 times and cells were analyzed by flow cytometry using the appropriate filters.

### Exposure of cells to magnetic nanoparticles

For our study, we used four different superparamagnetic iron-oxide preparations. Two agents used in laboratory setting only: CLIO-680, a fluorescent cross-linked iron oxide and MION-48, prototype monocrystalline iron-oxide; as well as two clinically approved agents: Ferumoxides (Feridex, Advanced Magnetics, USA) as a prototype clinical SPIO and one clinical approved USPIO: SHU 555C (Resovist S, Bayer Schering Pharma, Germany). The particle characteristics are summarized in Table 1. All particles display a similar type of dextran surface coating, although the density (polymer/Fe) differs. CLIO-680 has already been evaluated for targeting both human as well as murine monocyte subsets [8], [15], [22]. The two experimental agents (CLIO-680, MION-48) both fall into the USPIO category, similar to the clinical approved SHU 555C. As several studies have shown favorable uptake of SPIOs by monocytes as compared to USPIOs we additionally chose a clinically approved SPIO for labeling the monocyte subsets.

**Table 1:** Particle characteristics of the different iron-oxide nanoparticles.

Name	Characteristic	Mean Size (nm)	Coating	R1 (mM^−1^s^−1^)	R2 (mM^−1^s^−1^)
CLIO-680^19,20^	Experimental magnetofluorescent nanoparticle	29	Dextran	28.8 (0.47T/37°C)	74.3 (0.47T/37°C)
MION-48^22^	Experimental monocrystalline nanoparticle	26	Dextran	32.4 (0.47T/37°C)	130.5 (0.47T/37°C)
Ferumoxides (Feridex)^8^	Approved SPIO	80-150	Dextran	40.0 (0.47T/40°C)	160.0 (0.47T/40°C)
SHU 555C (Resovist S)^8^	Approved USPIO	21	Dextran	24.0 (0.47T/37°C)	60.0 (0.47T/37°C)

### CLIO-680

CLIO-680 are fluorescently labeled cross-linked iron-oxide nanoparticles used only in the experimental setting. Derived from MION-47, they have similar superparamagnetic characteristics as well as thick T10-dextran shell. In brief, MION particles are reacted with the crosslinking agent epichlorohydrin, which connects the partly free-floating dextran chains around the iron core. Thereby the iron core becomes completely caged which makes the particle chemically stable [23]. As the amino groups of the dextran chains become functionalized and nucleophilic they can be used for further conjugation, e.g. to fluorochromes or transfection agents such as HIV-tat peptides or protamine, as previously described [24]. To track the cellular processing of these nanoparticles we attached amine reactive near-infrared fluorochromes (VivoTag680, Perkin Elmer, Waltham, MA) to the nanoparticles. Each particle of CLIO-680 contained ∼11 molecules of dye and ∼80 free amine groups. We have successfully used CLIO-680 for targeting phagocytosis [8], [15], [22], by means of MRI as well as optical imaging [22].

### MION-48

MION-48, a prototype monocrystalline iron-oxide nanoparticle, is a further development of MION-47 with similar surface properties but higher relaxivities. The preparation used for the current experiments yielded a R2 relaxivity twice as high as CLIO-680, increasing the susceptibility in T2 weighted imaging. It is used in the experimental setting only; e.g. it has been successfully applied for in vivo monitoring of angiogenesis [25].

### Ferumoxides

Ferumoxides (Feridex I.V., Advanced Magnetics, USA) are colloid based SPIO that are taken up by the reticuloendothelial system and have been approved for liver imaging. The R1 and R2 relaxivities are 40.0 mM^−1^ s^−1^ and 160.0 mM−1 s−1 at 0.47T, respectively. Ferumoxides are a heterogeneous in the size with hydrodynamic diameters from 80 to 150 nm. They consist of non-stoichiometric magnetite cores, also covered with a dextran T-10 layer. Blood pool half-life of Ferumoxides after i.v. injection is about 6min. Ferumoxides are administered in patients at a dose of 838 µg Fe/kg [10].

### SHU 555C

SHU 555 C (Resovist S, Bayer Schering Pharma AG) are USPIO particles extracted from Ferucarbotran. The R1 and R2 relaxivities are 24.0 mM−1s−1and 60.0 mM^−1^s^−1^ at 0.47T in blood plasma at 40°C. The mean hydrodynamic size (including the hydrated dextran coating in an aqueous environment) is about 21 nm. SHU 555 C is administered in patients at a maximal dose of 2234 µg Fe/kg [10]. USPIOs have been successfully evaluated for the differentiation of metastatic versus benign/inflammatory lymph nodes [4], to characterize rupture prone atherosclerotic plaques [26] and to track neoangiogenesis in different murine tumor models [27].

FACS sorted monocytes or unsorted leukocytes were plated at 10^5^ cells/200 µl in a 96 well plate. Cell labeling with iron-oxide nanoparticles was performed at concentration from 0 to 2000 µg Fe/ml in 1640 RPMI for 2h, 37°C, humidified CO_2_ atmosphere. After the incubation period, cell suspensions were washed 3 times to separate labeled cells from unbound particles. For optical assessment by flow cytometry, cells were additionally stained with CD14 and CD16. After labeling, cells were counted again and cell viability was assessed with trypan blue. As controls, non-labeled FACS sorted monocyte subsets were used.

### Flow cytometry

Cell suspensions were stained with the following antibodies (all from BD Bioscience, unless otherwise stated) at a final concentration of 1∶100: CD11b-APC-Cy7/ICRF44, CD14-PE/M5E2, CD16 PE-Cy7/3G8, CCR2-Alexa-647/48607, CX3CR1-FITC/2A91 (MBL International, Woburn, MA), HLA-DR-APC/L243, CD163/GHI61, CD206/19.2, CD23/M-L233, CD120a/R1-B1. Cell phenotyping was performed using a LSRII Flow Cytometer (BD Bioscience, San Jose, CA) after appropriate compensations. For cell sorting, cells were labeled with CD14/CD16 and flow-sorted with a FACSAria (BD Bioscience, San Jose, CA). Purity of each monocyte subset population was ∼95% as determined by post-FACS flow-cytometric assessment. For the detection of fluorescent CLIO-680, the LSR II Flow Cytometer was equipped with a 685/LP and 695/40BP filter. Flow cytometric data were analyzed using FlowJo v.8.5.2 (Tree Star, Inc., Ashland, OR).

For morphologic characterizations, sorted cells were prepared on slides by cytocentrifugation (Shandon Inc., Pittsburgh, PA) at 10xg for 5 min, and stained with HEMA-3 (Fischer Scientific, Pittsburgh, PA).

### Fluorescence microscopy

FACS sorted human monocytes were immobilized by sedimentation for 1h on a poly-l-lysine coated coverslip (BD Biocoat, BD) and adherent cells were fixed for 30min in phosphate-buffered saline containing 3.7% paraformaldehyde (Sigma) and 0.2% Triton-X 100 (Sigma).

Cells were blocked for 30min in 1% BSA at 37°C and thereafter stained in blocking buffer supplemented with a FITC conjugated mouse anti-clathrin antibody (BD Transduction Laboratories) specific for the clathrin heavy chain at a dilution of 1∶200 for 1 h. The coverslips were washed three times in PBS and mounted onto a glass slide using 10 µl of mounting media (VECTASHIELD H-10000, Vector Laboratories). Fluorescence microscopy was performed on a Nikon Eclipse 80i microscope using a 60x/1.45 objective with oil immersion. For image acquisition a Hamamatsu C4742-95 digital camera connected to the IPLab (BD) software was used. Image analysis was performed using ImageJ v1.44 (http://rsbweb.nih.gov/ij/). Specifically differential CLIO-680 update was determined by assessing the mean grey values of the cytoplasm as a measure of CLIO-680 uptake of a least 10 cells per preparation in the Cy5.5-channel. Co-localization analysis of CLIO-680 and phagocytic vesicles was performed using the ImageJ plugin ‘Co-localization’.

### Spectrometry

Iron concentrations of FACS sorted monocyte subsets, labeled with the various iron-oxide nanoparticles, and non-labeled controls were quantified by atomic absorption spectrometry using a polarized Zeeman atomic absorption spectrometer (Z-8200, Hitachi, Japan). The cell pellets were dissolved with 1% sodium dodecyl sulfate (SDS) buffer. For Fe-measurements, the spectrophotometer was set to 248.3 nm and calibrated with six standards, containing 5000–200,019 µg/l Fe in 0.05 M HCl. For quality control, normal and abnormal lyphochek controls (Bio-Rad Laboratories, Munich, Germany) were used.

### MR imaging of labeled cells

FACS-sorted monocyte subsets were incubated with CLIO-680, MION-48, Ferumoxides and SHU 555C at 100 µg Fe/ml. 10^5^ labeled cells were resuspended in 300 µl sucrose gradient solution (Ficoll-Paque Plus, density 1.077 g/ml, GE Healthcare, NJ) to prevent sedimentation of cells during imaging [28]. The Ficoll-cell suspension in Eppendorf tubes was subsequently embedded in a water container, which minimizes susceptibility artifacts caused by interfaces with air or plastic. Unlabeled monocyte subsets were used as controls. MR imaging was performed on a 1.5T clinical imaging system (Achieva, Philips Medical Systems, Best, Netherlands) with a MAI wrist coil. For the simultaneous measurement of T1 and T2 the standard Philips MIX sequence was used. It consists of an interleaved IR and SE sequence with both signals being read out with multiple spin echoes. The echo trains serve to determine T2, while the ratios of the IR to SE signals lead to T1. The sequence and its subsequent data evaluation has been described in detail [29]. A 3D sequence with 3 slices was used to minimize artifacts caused by otherwise systematically smaller flip angles at the edges of 2D slices. Only the central slice was used for parameter quantization. Sequence parameters included TRIR 1500 ms, TI 100ms, TRSE 650 ms, TE 30*7ms, FOV 160×87 mm, matrix 192×104, slice thickness: 3 mm, in-plane resolution 0,83×0.83 mm, bandwidth 613 Hz/pixel, Acquisition time: 19:08 min.

### Statistical analysis

Data are expressed as Mean ± SD. Multiple comparisons of differences between the uptake of iron-oxide nanoparticles between the two monocyte subsets as well as differences between the nanoparticles were performed by analysis of variance (ANOVA). Statistical significance between the groups was calculated by the f-test. To evaluate differences in the expression of cell surface proteins before and after labeling, paired student's t-test was applied. A p-value <0.05 was considered statistically significant. Statistical analysis was performed with GraphPad Prism 5.0a for Mac (DraphPad Software Inc. San Diego, CA).

## Results

### Flow Cytometry and Fluorescence Microscopy

**Figure 1:**
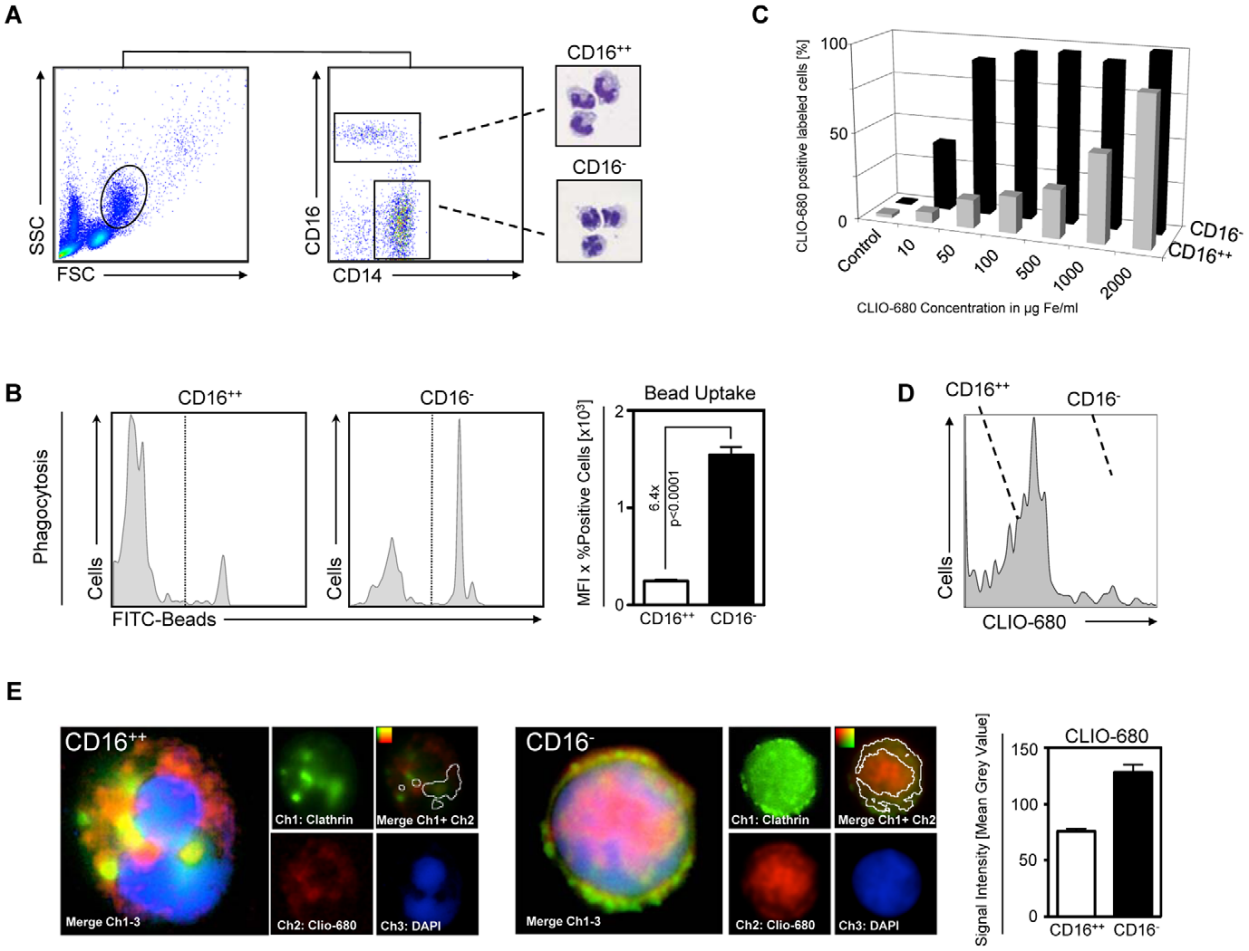
Monocyte subsets display different capacity of phagocytosis, which results in divergent uptake of iron-oxide nanoparticles. A) Flow cytometry dot plots of freshly isolated leucocytes from healthy donors. Monocytes are identified upon their FSC/SSC profile. Monocytes subsets are identified by their CD14/CD16 expression profile. H&E stains show monocyte morphology. B) Phagocytosis assay: flow cytometry histograms of both monocyte subsets after incubation with FITC-labeled latex beads for 2 h. Bar graph compares mean fluorescence intensity of internalized FITC-beads between both subsets, p-value shows significant difference (student's t-test). C) Bar graph depicts percentage of positively labeled alive monocyte subsets after 2h incubation with increasing concentrations of fluorescently labeled iron-oxide nanoparticles (CLIO-680), assessed by multi-color flow cytometry. D) Representative histogram shows fluorescence intensity of both monocyte subsets after labeling for 2 h with CLIO-680 at 100 µg Fe/ml. E) Quantitative immunofluorescence microscopy of FACS sorted monocytes after labeling with fluorescent iron-oxide nanoparticles (CLIO-680). Green: Clathrin, Red: CLIO-680, Blue: DAPI/nuclear staining. For co-localization analysis (upper right panel) images were taken under reproducible conditions and no additional enhancement was performed for the red and green channels. Fiducials indicate the red-green signal intensities used for determination of co-localization. White-circled areas indicate co-localization of Clathrin and CLIO-680 signal. Bar graph compares mean grey values of intracellular CLIO-680 signal between both subsets (from n = 3 different experiments with >10 of each subset analyzed per experiment).

In a first set of experiments we identified two major monocyte subpopulations by flow cytometry and assessed their phagocytosis capacity with both fluorescent beads as well as fluorescent nanoparticles. Human monocytes were obtained from peripheral blood of healthy volunteers by density-gradient centrifugation. The procedure enriches mononuclear cells by removing neutrophils and other granulocytes. Monocytes (10–30 µm) are larger than lymphocytes (5–15 µm) and can be identified by a distinct forward scatter/side scatter profile (FSC/SSC) [8], [13], [21]. With respect to the expression of CD14 and CD16, monocytes can be divided into two major populations: CD14^++^CD16^-^ monocytes, which make up about ∼85% of all monocytes, and a minor population of CD14^+^CD16^++^ monocytes (Figure 1A). Upon 2h incubation with fluorescent latex-beads, CD14^++^CD16^-^ monocytes display a significant higher bead uptake, assessed by the mean fluorescent intensity (MFI), indicating a higher phagocytosis capacity of the CD14^++^CD16^-^ monocytes as compared to their CD14^+^CD16^++^ counterparts (Figure 1B). Subsequently we investigated the uptake of the fluorescent superparamagnetic nanoparticle CLIO-680 by flow cytometry at different particle concentrations for 2h. At concentrations from 10–1000 µg Fe/ml a significantly higher proportion of CD14^++^CD16^-^ monocytes is labeled with CLIO-680 as compared to the CD14^+^CD16^++^ monocytes (Figure 1C). Only at concentrations of 2000 µg Fe/ml CD14^+^CD16^++^ monocytes are positively labeled at a comparable degree. However, labeling concentrations above 100 µg Fe/ml are entirely unphysiologic when compared to plasma concentration of iron-oxide nanoparticles after i.v. administration [10]. Therefore, subsequent labeling experiments were performed at iron concentrations of 100 µg Fe/ml. At 100 µg Fe/ml cell viability was ≥98%, as determined by trypan blue staining. This is in accordance with previous labeling studies that demonstrated that only at concentrations >500 µg Fe/ml iron exerts relevant toxicity to the incubated cells [10], [11]. At 100 µg Fe/ml the two subsets populations can be clearly discriminated from each other by a different labeling efficiency (Figure 1D). The mechanism of particle internalization is directly related to particle size. Particles <200 nm are internalized via endocytosis and transported intracellularly within clathrin-coated vesicles [30]. After labeling FACS sorted monocyte subsets with fluorescent nanoparticles, fluorescence of nanoparticles co-localized with clathrin fluorescence, indicating a clathrin dependent intracellular storage of the superparamagnetic particles (Figure 1E).

### Spectrometry

**Figure 2:**
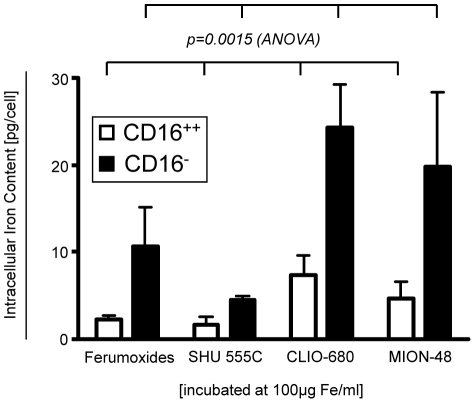
Absolute quantification of intracellular iron content in monocyte subsets by Zeeman atomic emission absorption spectrometry. FACS sorted monocyte subsets were incubated with four different iron-oxide nanoparticles (Ferumoxides, SHU 555C, CLIO-680, MION-48) at 100 µg Fe/ml for 2 h. Differences in intracellular iron contents between the subsets were assessed by ANOVA. P-values indicate significant differences in intracellular iron-content between the monocyte subsets. n = 3-5 for each bar graph. Iron levels of non-labeled control monocytes were below the detection limit.

To quantify the nanoparticle uptake, FACS sorted monocyte subsets were incubated with different nanoparticles at concentration of 100 µg Fe/ml. As particle uptake is both related to particle size and surface coating we studied the particle uptake of two experimental and two clinically approved iron-oxide particles (Table 1). The amount of intracellular iron, representative for particle uptake, was significantly higher in CD14^++^CD16^-^ monocytes (p = 0.0015, ANOVA) as compared to the CD14^+^CD16^++^ monocytes (Figure 2). Intracellular iron-concentrations were significantly different with regard to the labeling particle (p = 0.0251, ANOVA), with both CLIO-680 and MION-48 showing a higher uptake as compared to the two clinical approved Ferumoxides and SHU 555C. Iron-levels of non-labeled monocytes were below the detection limit (of 100 ng Fe/ml cell suspension).

### MR imaging

**Figure 3:**
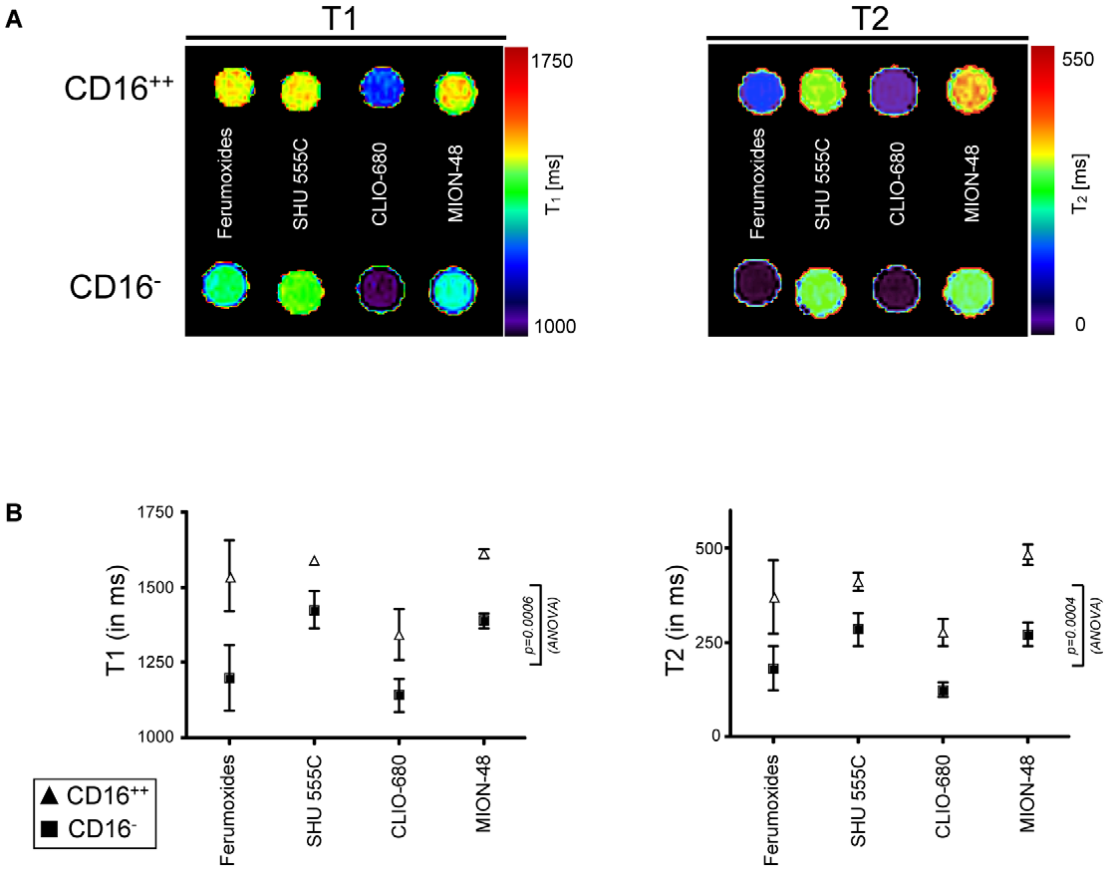
Different degree of labeling with variant iron-oxide nanoparticles results in different alteration of T1 and T2 relaxation rates between the monocyte subsets. A) Representative examples of phantom imaging of FACS sorted nanoparticle-labeled monocyte subsets with a clinical 1.5T MRI system. Phantom plots show NIH-color coded transversal sections through the samples tubes containing the labeled monocyte subsets embedded in Ficoll solution to prevent sedimentation during imaging. B) Graphs show corresponding T1 and T2 relaxation times obtained from phantom experiments (graphs show mean ± SD from 3 different experiments). After labeling with variant iron-oxide nanoparticles (Ferumoxides, SHU 555C, CLIO-680, MION-48) at 100 µg Fe/ml for 2h, the CD16^-^ subset is characterized by shorter T1 (p = 0.0006, ANOVA) and T2 (p = 0.0004, ANOVA) relaxation times as compared to CD16^++^ monocytes. Mean relaxation times of non-labeled controls were as followed: T1 = 2190±43 ms/2173±49 ms (CD16^++^/CD16^-^), T2 = 841±28 ms/835±30 ms (CD16^++^/CD16^-^).

To determine if the different cellular uptake of nanoparticles between CD14^++^CD16^-^ and CD14^+^CD16^++^ monocytes translates to imageable differences, we incubated FACS sorted monocytes with the various nanoparticles for subsequent ex vivo MR imaging (Figure 3). Increased nanoparticle uptake of the CD14^++^CD16^-^ monocytes indeed resulted in significant lower T1 (p = 0.0006, ANOVA) and T2 (p = 0.0004, ANOVA) relaxation times for the various particles. Also the particle type had impact on the T1 (p = 0.006, ANOVA) and T2 (p = 0.0025, ANOVA) relaxation times, with both Ferumoxides and CLIO-680 leading to a more severe drop in T1 and T2 relaxation time as compared to SHU 555C and MION-47. Mean relaxation times of non-labeled controls were as followed: T1 = 2190±43ms/2173±49ms (CD16^++^/CD16^−^), T2 = 841±28ms/835±30ms (CD16^++^/CD16^−^).

### Phenotype alterations after cell labeling

**Figure 4:**
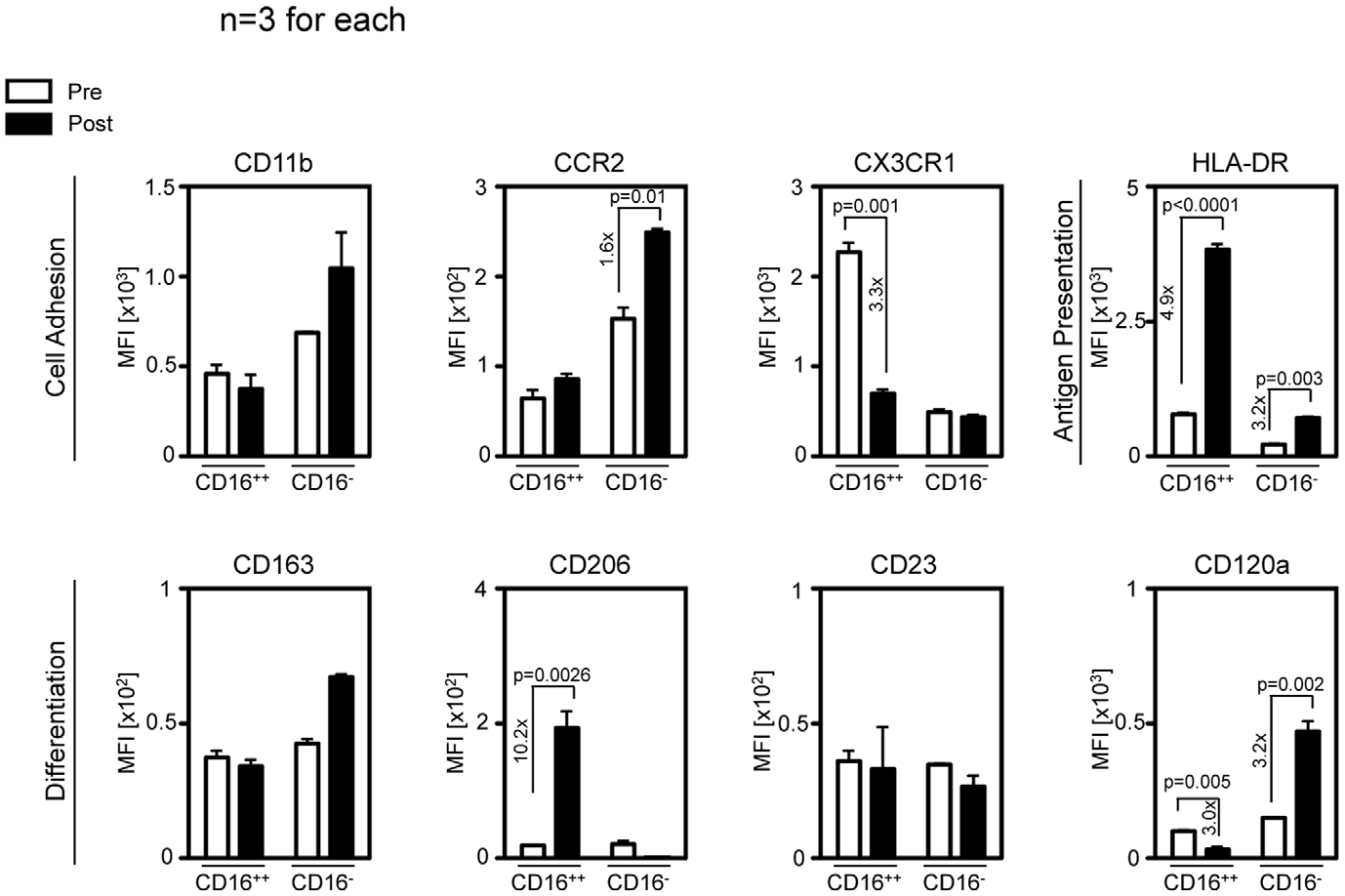
Alteration of specific phenotypic markers after incubation with fluorescent iron-oxide nanoparticles compared between the CD16^-^ monocyte and the CD16^++^ monocyte. Sorted monocytes were incubated with CLIO-680 at 100 µg Fe/ml for 2h and subsequently analyzed by flow cytometry. Markers important for monocytes to enter the target tissue (CD11b, CCR, CX3CR1), to differentiate (CD23, CD163, CD120a, CD206) and to present antigens (HLA-DR) are investigated. Mean fluorescence intensities of recorded events are shown ± SD (from 3 different experiments). Statistically significant up or down regulation of surface proteins, if present, is indicated within each graph (paired students t-test).

An important aspect of nanoparticle uptake by mononuclear phagocytes is whether the cell labeling affects their phenotype. Therefore we assessed a preliminary set of cell surface markers before and after incubation with the fluorescent nanoparticle CLIO-680 to search for potential alterations of the cell phenotype due to the labeling process. We studied the expression of cell adhesion molecules (CD11b, CCR2, CX_3_CR1), HLA-DR as marker for antigen presentation as well as markers expressed during the differentiation of monocytes to macrophages (CD23, CD163, CD206). Further, CD120a, the TNFα receptor was analyzed as a marker indicating the susceptibility of monocytes to inflammatory stimuli. After 2 h of incubation with CLIO-680 at 100 µg Fe/ml CD14^++^CD16^-^ monocytes selectively up regulated the CCL2 receptor while CD14^+^CD16^++^ monocytes down regulated the fractalkine receptor CX_3_CR1. Both monocyte subsets up regulated HLA-DR indicating an increased potential to present processed antigens on their cell surface. In terms of differentiation, CD14^+^CD16^++^ monocytes up regulated CD206, while CD23 and CD163 showed no marked difference. While CD120a expression was down regulated in CD14^+^CD16^++^ monocytes, it was expressed at significantly higher levels in CD14^++^CD16^-^ monocytes after labeling with CLIO-680 (Figure 4).

## Discussion

To non-invasively study disease mechanisms by means of Magnetic Resonance Imaging, labeling of human monocytes with both positive contrast agents as well as superparamagnetic nanoparticles has been performed [10], [28], [31]. Ex vivo labeled human cells can be successfully reinjected into patients and tracked with MRI [32]. The ex vivo labeling of human phagocytes with subsequent reinjection and in vivo MR Imaging is expected to enhance inflammatory or neoplastic lesions more specifically than the pure contrast agent after i.v. injection. Reinjection of ex vivo labeled monocytes could direct the contrast agents more specifically to the pathologic target. This could solve current problems of limited sensitivity of MR imaging and increase contrast-to-noise ratio due to a limited background signal. Up to now, ex vivo labeling of human monocytes has been performed unselectively without respect to their heterogeneity [10], [11]. However, as recent work on monocyte heterogeneity has revealed monocyte subpopulations that perpetuate disease and promote inflammation, while other monocyte subsets promote healing and resolve inflammation [1], [2], [14], [15], selective labeling of these subsets will be important for targeting specific disease mechanisms by MRI.

We have previously shown that human monocyte subsets display different capacity of phagocytosis [8] and these results were recently confirmed by Cros et al. [33]. Here we further exploit these properties and show that CD14^++^CD16^-^ monocytes show a higher uptake of both experimental and clinically approved iron-oxide contrast agents and that this increased uptake leads to shorter T1 and T2 relaxation times at 1.5T. The iron uptake of the two clinically approved iron-oxide nanoparticles, quantified by Zeeman spectrometry, was comparable to the values reported by Metz et al [10]. The cellular uptake of MION-48 by both monocytes subsets was higher than the previously reported uptake of MION-46 by cultured peritoneal macrophages [34], which is attributed to the variant differentiation stage of the phagocytes as well as different particle characteristics of MION-46 versus MION-48. In general, differences between the different agents are attributed to particle size and surface coating, opsonization as well as surface charge [10], [35].

Additionally intracellular particle compartmentalization influences the relaxivity. Therefore no linear relationship can be assumed between the amount of particle uptake and relaxivity [9]. Using a magnetofluorescent nanoparticle we demonstrate that the internalized iron-oxide particle (CLIO-680) co-localizes with clathrin-coated vesicles. Also in our study the amount of intracellular nanoparticles measured by atomic absorption spectrometry did not directly translate to the observed relaxation times. As all of the four particles tested were similarly dextran coated, this is mainly attributed to particle size, opsonization and cellular compartmentalization. Several studies have demonstrated that endocytosis of nanoparticles is highly dependent on the particle size [36]. Regarding the particle size two important aspects have to be considered. First, there is an inherent polydispersity within any given batch of nanoparticles and some nanoparticle formulations, e.g. Ferumoxides are quite heterogeneous in size. Secondly, although nanoparticles retain a certain size after synthesis, they may aggregate during in-vitro and in-vivo studies into fairly different shapes and sizes that may further alter the endocytosis of the particle. Differences in opsonization also have to be taken into account when phagocytosis of nanomaterials by the targeted cells is discussed, however only preliminary data exist on how the complement system interacts with superparamagnetic nanoparticles. In this context Beduneau et al. demonstrated that altering opsonization by coupling of IgG to the surface of SPIOs increased the particle uptake by 10x with subsequently improved enhancement of lymphoid tissue by high-field MRI [37]. Surface charge of the particles is another factor influencing the interaction with cell-surface proteins and further uptake. The surface charge is mainly dependent on the coating material, in our case T10 dextran. Therefore different surface charge is not seen as major reason for the different cellular uptake in our study. Yet it is known that functional groups attached to the nanoparticle surface can further modify nanoparticle properties [36]. Thus, it cannot be excluded that the attachment of the NIRF fluorochrome to the CLIO particles in our study alters the particle internalization and compartmentalization. This may explain the fact that compared to MION-48 the uptake CLIO-680 is slightly higher and that the decrease in T1 and T2 relaxivity of CLIO-680 labeled monocyte subsets is markedly higher as compared to MION-48.

The results obtained in this study can not be necessarily expanded to particles with other surface coatings. The different surface properties, e.g. of polyvinyl alcohol or dopamine-polyethylene-glycol coating is known to alter the cell-particle interaction and subsequent internalization of the particle [36]. However for all particles investigated, CD14^++^CD16^-^ monocytes showed higher intracellular iron concentrations compared to CD14^+^CD16^++^ monocytes and T1 and T2 relaxation times were lower for all tested agents in CD14^++^CD16^-^ monocytes compared to their CD14^+^CD16^++^ counterparts.

As previous studies in humans have demonstrated, blood levels of CD14^+^CD16^++^ monocytes are elevated in chronic diseases. The reason for the elevated CD14^+^CD16^++^ levels in these inflammatory conditions is not entirely clear yet. However previously published work suggests that the CD14^+^CD16^++^ monocytes are active in production of pro-inflammatory cytokines (e.g. TNFα and IL1β) and chemokines (e.g. CCL3) and exhibit a higher potency in antigen presentation, whereas the CD14^++^CD16^-^ monocytes with increased phagocytosis capacity are involved in tissue repair [33], [38].

Labeling of monocytes with iron-oxide based nanoparticles may affect their phenotype as well as certain functional properties. Oude Engberink studied the migratory capacity after labeling of monocytes with SPIOs and USPIOs as well as production of certain cytokines, such as IL1 and IL6. Neither the migratory capacity, nor the production of the investigated cytokines was affected by the labeling procedure, assessed directly after incubation [11]. Yet, changes of the cellular phenotype may still occur. Additional to the uptake experiments performed we analyzed a preliminary set of cell surface markers before and after labeling the cells with iron-oxide nanoparticles. Although the cell labeling procedure consumes a limited amount of time, in our case 2h, both the artificial labeling environment as well as the particle endocytosis itself may significantly affect the expression of cell surface proteins. Indeed we observed significant changes in the expression in some of the markers tested. Remarkably, changes in the expression of cell adhesion molecules were observed. While CD14^++^CD16^-^ monocytes up regulated the CCL2 receptor, CD14^+^CD16^++^ monocytes down regulated the fractalkine receptor CX_3_CR1. Both molecules are important for monocytes to adhere to the endothelium and extravasate into the target tissue [39], [40]. An up regulation of these adhesion molecules may facilitate tissue entry while down regulation may aggravate it. Both monocyte subsets increase their expression of HLA-DR after incubation with iron-oxide nanoparticles, which indicates a shift towards antigen presenting cells. This is not surprising, as phagocytosis is regularly followed by increased antigen presentation [41]. However a shift of monocytes towards APC induced by the labeling procedure may change the physiologic course, monocytes take on their route to tissue repair and homeostasis and prime the labeled cells towards a certain commitment. Increased expression levels of the mannose receptor CD206, which is an important mediator of macrophage cell migration, demonstrates a selective differentiation of CD14^+^CD16^++^ monocytes towards the macrophage lineage already after two hours of in-vitro cell labeling, while CD14^++^CD16^-^ monocytes did not alter their expression of CD206. Up regulation of the TNFα receptor CD120a in CD14^++^CD16^-^ monocytes during the labeling procedure makes the CD14^++^CD16^-^ monocytes more susceptible to inflammatory stimuli mediated by TNFα. Although this set of phenotypic markers only is a very limited selection, the current results demonstrate that even the short ex vivo labeling procedure alters the phenotype of incubated naïve monocytes and, that the up or down regulation of these markers differs between the two main subsets.

The revealed differential endocytosis capacity of the monocyte subsets has diagnostic potential, which goes beyond the previous published work on unselective labeling of monocytes. Monocyte subsets continue to emerge as important mediators of inflammation and tissue homeostasis with certain subsets attenuating disease, others promoting inflammation [1], [2], [38]. Targeting of certain inflammatory diseases with re-injected ex vivo labeled monocytes has been proposed as a novel non-invasive diagnostic tool. Both the efficiency as well as the specificity will be increased when only specific monocyte subsets are used, that are particularly involved in the disease process and thus preferentially recruited to the site of inflammation. Therefore the labeling protocols will have to be optimized for the specific monocyte subsets. To take advantage of labeled monocyte subpopulations as more specific disease reporters, the detailed roles of monocyte subsets in certain diseases has be to further elucidated.

In addition to their diagnostic potential, monocytes loaded with certain nanoparticles have been proposed as potential carriers to enhance drug delivery [37]. Monocytes that incorporated nano-sized drug-carriers could act as Trojan horses by crossing e.g. the blood-brain barrier and enabling drug delivery [42]. In this context it may also be favorable to use specific monocyte subsets that achieve a higher nanoparticle load and exhibit a facilitated tissue-entry to the target site.

The current study investigated the uptake of various iron-oxide nanoparticles by monocyte subsets from healthy humans as well as phenotypic changes induced by the labeling procedure. Further labeling experiments will have to focus on labeling monocytes from diseased patients. Especially in patients with certain inflammatory conditions such as atherosclerosis, multiple sclerosis or infectious diseases, activated monocytes may significantly alter their endocytosis capacity resulting in different loading capacities. Additionally, the specific roles of monocyte subsets in these diseases has be further investigated to being able to use monocyte subsets as imaging reporters in clinical radiology.

In summary, this study shows that the two main subsets of human monocytes differentially take up iron-oxide based nanoparticles which leads to different T1 and T2 relaxation times when the cells are investigated ex vivo. The specific labeling of the human subtypes of mononuclear phagocytes will have impact on both experimental and clinical trials working with cell labeling of human phagocytes.
